# Brain tumours and cigarette smoking: analysis of the INTERPHONE Canada case–control study

**DOI:** 10.1186/1476-069X-13-55

**Published:** 2014-06-27

**Authors:** Stephen Vida, Lesley Richardson, Elisabeth Cardis, Daniel Krewski, Mary McBride, Marie-Elise Parent, Michal Abrahamowicz, Karen Leffondré, Jack Siemiatycki

**Affiliations:** 1 Centre de Recherche, Centre hospitalier de l’Université de Montréal (CHUM), Montréal, Québec, Canada; 2 McGill University, Montréal, Québec, Canada; 3 Centre for Research in Environmental Epidemiology (CREAL), Municipal Institute of Medical Research (IMIM), CIBERESP, Barcelona, Spain; 4 McLaughlin Centre for Population Health Risk Assessment, University of Ottawa, Ottawa, Canada; 5 British Columbia Cancer Agency, Vancouver, Canada; 6 INRS-Institut Armand-Frappier, Université du Québec, Laval, Québec, Canada; 7 University of Bordeaux, ISPED, Centre INSERM U897-Epidemiology-Biostatistics, Bordeaux, France; 8 Guzzo-SRC Research Chair in Environment and Cancer, Centre de Recherche, Centre hospitalier de l’Université de Montréal (CHUM), 850, rue St-Denis, room S02-458, Montreal, Quebec H2X 0A9, Canada

**Keywords:** Glioma, Meningioma, Brain tumours, Cigarette, Smoking, Cigarette smoking

## Abstract

**Background:**

There is conflicting evidence regarding the associations between cigarette smoking and glioma or meningioma. Our purpose is to provide further evidence on these possible associations.

**Methods:**

We conducted a set of case–control studies in three Canadian cities, Montreal, Ottawa and Vancouver. The study included 166 subjects with glioma, 93 subjects with meningioma, and 648 population-based controls. A lifetime history of cigarette smoking was collected and various smoking indices were computed. Multivariable logistic regression was used to estimate odds ratios (ORs) between smoking and each of the two types of brain tumours.

**Results:**

Adjusted ORs between smoking and each type of brain tumour were not significantly elevated for all smokers combined or for smokers with over 15 pack-years ((packs / day) x years) accumulated. We tested for interactions between smoking and several sociodemographic variables; the interaction between smoking and education on glioma risk was significant, with smoking showing an elevated OR among subjects with lower education and an OR below unity among subjects with higher education.

**Conclusion:**

Except for an unexplained and possibly artefactual excess risk in one population subgroup, we found little or no evidence of an association between smoking and either glioma or meningioma.

## Introduction

The incidence of primary intracranial tumours in Western Europe, North America, and Australia ranges from 4 to 11 per 100,000 population per year. This is approximately four times the incidence reported in the lowest risk regions of the world, although some of this variability may be due to differences in access to diagnostic services [1,2]. In the United States the age-adjusted incidence increased by about 19% in men and 27% in women between 1973 and 2003 [1].

Glioma and meningioma are the two most common types of brain tumours, comprising approximately 75% of all brain tumours [1-3]. Gliomas are more common in men; in the US, they are more common among whites than among blacks and Hispanics [1]. The median age at diagnosis is 53 years [1]. Due to the relatively young age at presentation and poor prognosis, gliomas account for a substantial number of years of life lost. While some forms of glioma are associated with specific genetic mutations or inherited syndromes [2,3], such as neurofibromatosis type 1 and 2 [2], the only modifiable risk factor unequivocally associated with glioma is high dose therapeutic ionizing radiation [2,3]. Meningioma, less common than glioma, is more common in women than men and has a later median age at diagnosis of 64 years [1]. Like glioma, meningioma is associated with ionizing radiation and with neurofibromatosis type 2 (NF2) [4]. A history of tuberous sclerosis has also been reported to increase the risk of brain tumour [1]. For the most part, the etiology of brain tumours is not understood; in particular it is not known if exogenous chemicals are capable of causing human brain tumours.

Cigarette smoking is a major source of multiple systemically absorbed chemical carcinogens including, among others, polycyclic aromatic hydrocarbons (PAHs) and *N*-nitroso compounds. Among smokers, tobacco smoke is by far the greatest source of exposure to *N*-nitroso compounds [5]. These potent agents are associated with tumours of the nervous system in animals, whether administered transplacentally [6] or intravenously [7,8]. Although nicotine may increase the permeability of the blood brain barrier [9], it is unknown if *N*-nitroso compounds penetrate to human brain tissue [10].

From a public health perspective, cigarette smoking is the single most important cause of human cancer. In high-income countries, tobacco smoking is responsible for 25 – 30% of overall cancer mortality [11]. Smoking is associated with elevated rates of cancer of the lung, bladder, cervix, colon and rectum, oesophagus, kidney, larynx, mouth and throat, pancreas, stomach, nasal cavity, liver, ovaries, and with acute myelogenous leukemia [12]. Because tobacco smoking causes tumours at multiple sites, it is conceivable that smoking could affect brain tumour risk. If smoking were shown to be a risk factor, it would constitute an important “proof of principle” that exogenous chemicals are capable of causing human brain tumours.

Most case–control studies have not shown increased risk of glioma in relation to tobacco smoking [5,13-20]; others found elevated relative risks that were not statistically significant or confined to subgroups of the population studied [21-24]. Among cohort studies, some found no association [25-27] and some found significant positive associations in women but not men [28] and in a study of women only [29]. A recent meta-analysis on smoking and glioma found no overall association when case–control and cohort studies were pooled, although there was a small but significant increased risk among cohort studies [30]. For meningioma, some case–control studies found no evidence of increased risk [16,27,31,32] and others found an association in one sex but not the other [17,18,33-35]. Two cohort studies did not find elevated risk [25,27]. A review concluded that the evidence regarding smoking in relation to glioma and meningioma is largely null, but still inconclusive [1]. A more recent large prospective study found no association between smoking and carcinomas of the brain [36]. Finally, a recent comprehensive evaluation of the cancer risks associated with smoking conducted by the International Agency for Research on Cancer did not list the nervous system as a target organ for tobacco-related cancer in humans [37].

Because of the rarity of brain tumours and the difficulty of mounting large studies, it is important to increase the number of good quality studies in the expectation that true patterns will emerge from the juxtaposition of a large number of published results. The present analysis of the smoking data from a Canadian case–control study of brain tumours is intended to contribute to the growing body of scientific evidence on which a more complete evaluation of the association between smoking and brain tumour risk can be based.

## Methods

### Design

The INTERPHONE study is an international consortium of case–control studies of glioma, meningioma, acoustic neurinoma and parotid gland tumours, with the primary purpose of assessing mobile phone use as a possible risk factor. Its methodology has been described in detail elsewhere [38]. Sixteen centres in 13 countries used a common core protocol. Three Canadian centres participated, one each in Montreal, Ottawa and Vancouver. The present report derives from the Canadian component of the INTERPHONE study.

### Subjects

Because of the emphasis on the use of mobile telephones as a possible risk factor for brain cancer, the study focused on relatively young people. Eligible cases were males and females aged 30 to 59 years residing in the study regions and diagnosed during the study period with a confirmed first primary glioma or meningioma. Montreal subjects were also required to be Canadian citizens. Recruitment took place between 2002 and 2004. All diagnoses were histologically confirmed or based on unequivocal diagnostic imaging. Each centre established procedures for the rapid ascertainment of cases from their respective study bases. Close relationships with hospital-based tumour registries, diagnostic and treatment units and medical records departments were maintained so that cases were not missed and necessary authorizations to contact cases were obtained. Since cases in Vancouver were ascertained via a provincial cancer registry and cases in Montreal and Ottawa by reviewing files of all major diagnostic facilities, it is reasonable to assume that we ascertained upwards of 90% of all eligible cases of glioma.

Sampling of controls varied among centres. Sources of controls were: electoral lists in Montreal, random-digit dialling in Ottawa, and the British Columbia provincial health insurance agency in Vancouver. In each centre, controls were selected to approximate the age and sex distribution of the cases series, in Ottawa and Vancouver by using individual matching and in Montreal by stratified sampling.

The study was approved by the research ethics boards of all the centres involved. All cases for whom physician authorization had been obtained and all controls were informed about the study and asked to participate. Written informed consent was obtained from all participating subjects.

### Data collection and variable definition

Information on exposure and covariates was gathered by a trained interviewer using a computerized questionnaire during interviews, mainly face-to-face, with the study subject or a proxy. The questionnaire included items on socio-demographic factors, mobile phone use, cigarette smoking, occupational exposure to electromagnetic fields, and medical history, including medical exposure to ionizing radiation. Demographic data included birth date, sex, address and highest attained education, coded in five categories. Using subjects’ addresses and postal codes, we were able to derive the median household income of the subject’s area of residence, as obtained from Canadian census data. Smoking data collected included history of ever regular smoking (defined as daily smoking for six months or more), including age at which regular smoking started, age at cessation, periods of abstinence, and average amount smoked during each smoking period. Cases and controls were assigned a reference date, namely, the date of diagnosis for cases and the date of interview for controls. Because smoking habits often change during periods of illness even prior to diagnosis and because of the long latency between exposure to tobacco smoke and tumour diagnosis, tobacco consumption within two years of the reference date was discounted. Questionnaires included items on possible risk factors for brain tumour such as neurofibromatosis, tuberosclerosis, and prior therapeutic radiation. Family history of primary brain tumour in first-degree relatives was also examined.

### Data analysis

Notwithstanding the fact that controls were selected separately for each type of brain tumour, and that there were individual matching algorithms in two of the centres, for the present analysis, each case series was compared with the entire pool of eligible interviewed controls. This was done for several reasons. First, the low response rates among controls in particular would have led to elimination of many subjects and significant loss of information had we insisted on maintaining the individual matching when analysing the results. Second the matching criteria, age and sex, do not provide close covariate control, like neighbours or friends might. Third, the main limitation of this study is statistical power, and the inclusion of the largest possible set of controls maximizes the power. This strategy led to the two sets of results (glioma and meningioma) not being independent of each other because of the common control series. However, it was felt that the efficiency and statistical power gain from this double use of the controls was more important than the maintenance of statistical independence between the two sets of results. Unconditional multivariable logistic regression was used to examine the relationship between case–control status and smoking. Three smoking metrics were modelled, each in a separate model: ever/never regular smoking, duration of smoking in years (nonsmokers ; 1–20 years; >20 years) and pack-years of cigarettes smoked (nonsmokers; 1 – 15 pack-years (packs per day x years); >15 pack-years). These cutpoints were selected in order to give approximately equal sample sizes in each group. The following covariates were included: age (30–39, 40–49 and 50–59 years), sex, education (lower defined as completion of high school or less and higher defined as any post-secondary education), and study region (Montreal, Ottawa or Vancouver). While we derived the median census tract income of the neighbourhood of residence, this variable was not included in the models because it was highly correlated with education and the small number of subjects contra-indicated the loss of additional degrees of freedom. Also, we did not include variables for cellphone usage because that has not been recognized as definitely carcinogenic, and to the extent that there is any evidence of risk, it is only in a small fraction of users and the corresponding relative risk estimates are very low. In addition to the overall study sample analyses, we conducted various subgroup analyses by sex and other characteristics.

Following the observation of some differences in findings between men and women, we decided to explore possible effect modification between smoking (ever/never) and each of the following covariates: sex, age decade as described above, education level as described above, and study centre. We examined these interactions using logistic regression with backward elimination, removing the least statistically significant interaction in each step. We did not adjust the alpha level for multiple comparisons.

## Results

There were 271 eligible gliomas, 133 eligible meningiomas and 1,325 eligible controls. Successful interviews were obtained for 166 (61.3%) gliomas, 93 (69.9%) meningiomas and 648 (48.9%) controls. Response rates were rather similar for males and females. Table 1 summarizes the reasons for non-participation.

**Table 1:** Response rates and reasons for non-response among brain cancer cases and controls in Canada

**Response**	**Glioma cases**		**Meningioma cases**		**Controls**	
	**N**	**%**	**N**	**%**	**N**	**%**
Eligible	271	100.0	133	100.0	1325	100.0
Responded	166	61.3	93	69.9	648	48.9
Non-response						
*Subject refused*	20	7.4	17	12.8	428	32.3
*Physician refused*	19	7.0	10	7.5	-	-
*Subject deceased or ill, no proxy available*	48	17.7	3	2.3	6	0.5
*Untraced or other*	18	6.6	10	7.5	243	18.3

Given the somewhat low participation rates, there was some concern about possible biases due to unrepresentative participants. In order to detect possible self-selection bias in respondents, we instituted a policy of asking controls who refused to participate in the lengthy interview if they would agree to answer a 5-minute questionnaire that included a question on attained education level. Among the 108 nonparticipant controls who agreed to provide such information, the percentage who had greater than high school education was 50.9%, whereas among the 648 participant controls in the main study, the percentage was 71.3%. To the extent that the nonparticipants who agreed to answer the brief questionnaire were representative of all nonparticipants, this indicates that participants in the main study had higher education than nonparticipants, at least among controls. As smoking is often inversely related to education level, this raises the possibility of confounding by educational attainment and highlights the importance of controlling for education level in analyses of smoking as a risk factor. Unfortunately we had no information on smoking habits of non-participants.

Table 2 presents selected demographic data for participating cases and controls. As expected, males predominated among glioma cases and females among meningioma cases. Cases had slightly lower education level and slightly lower median census tract income than controls. Glioma cases tended to be younger than controls and meningioma cases tended to be older than controls, as expected. While there were some proxy respondents among cases, there were hardly any for controls. Proxies were usually the surviving spouse or offspring. In order to assess whether this may have biased the findings, we reanalysed the study data, excluding proxy responses altogether. These results were very close to the ones in the Tables.

**Table 2:** Selected socio-demographic characteristics of participating cases and controls

**Characteristic**	**Glioma cases N = 166**	**Meningioma cases N = 93**	**Controls N = 648**
**Region, %**			
Montreal	39.2	50.5	36.0
Ottawa	13.9	16.1	27.3
Vancouver	47.0	33.3	36.7
**Male sex (%)**	63.3	28.0	48.9
**Age, %**			
30-39 years	24.7	9.7	19.0
40-49 years	32.5	32.3	33.2
50-59 years	42.8	58.1	47.8
**Education > high school, %**	61.4	54.8	71.3
**Census tract median household income, C$, mean (standard deviation (sd))**	46,557 (16,287)	44,261 (16,687)	47,302 (17,350)

There were very few subjects with neurofibromatosis, tuberous sclerosis, history of therapeutic radiation, or family history of primary brain tumour in first-degree relatives. These variables were therefore not included in smoking analyses.

Table 3 presents lifetime smoking patterns among cases and among controls, by sex. The lifetime prevalence of smoking and the cumulative pack-years of smoking were somewhat higher among males than females. Crude differences in smoking parameters between cases and controls were rather small; among males the smoking rates were slightly higher among glioma and meningioma cases than among controls, whereas smoking rates in females were slightly lower among glioma cases than among controls. Smoking was associated with sex, with age and with education. For male controls, 64% of subjects in the lower education stratum were ever regular smokers and the smokers had a mean of 28.5 pack-years whereas 53% of subjects in the higher education stratum were ever regular smokers and those smokers had a mean of 18.9 pack-years. For female controls, 56% of subjects in the lower education stratum were ever regular smokers and the smokers had a mean of 20.4 pack-years whereas 45% of subjects in the higher education stratum were ever regular smokers and those smokers had a mean of 14.0 pack-years.

**Table 3:** Comparison of smoking exposure measures between cases and controls, stratified by sex

**Smoking exposure**	**Glioma cases**	**Meningioma cases**	**Controls**
**Males**	**N = 105**	**N = 26**	**N = 317**
Ever regular smokers, %	61.0	65.4	55.8
Current smokers, %	26.7	46.2	24.6
Ex-smokers, %	34.3	19.2	31.2
Smokers only	N = 64	N = 17	N = 177
Cig/day, mean (sd)	20.4 (13.8)	20.5 (12.4)	19.7 (12.8)
Duration, mean years (sd)	18.9 (11.1)	21.7 (11.5)	20.6 (11.4)
Pack-years, mean (sd)	21.9 (21.4)	21.9 (17.0)	21.9 (19.8)
**Females**	**N = 61**	**N = 67**	**N = 331**
Ever regular smokers, %	39.3	50.7	48.3
Current smokers, %	19.7	20.9	19.9
Ex-smokers, %	19.7	29.9	28.4
Smokers only	N = 24	N = 34	N = 160
Cig/day, mean (sd)	17.2 (10.1)	18.8 (13.2)	15.6 (11.7)
Duration, mean years (sd)	18.9 (10.3)	20.1 (10.8)	18.7 (11.2)
Pack-years, mean (sd)	18.5 (18.3)	20.9 (18.5)	16.2 (16.6)

Table 4 presents adjusted odds ratios (ORs) and 95% confidence intervals (CIs) between various smoking metrics and brain tumours. Because smoking behaviour, glioma incidence and meningioma incidence differ between males and females, results are presented for males and females both separately and together. The overall OR between ever regular smoking and glioma was 0.96 (95% CI: 0.67 – 1.38) and that between smoking and meningioma was 1.09 (95% CI: 0.69 – 1.72). ORs were higher among males than females. Nevertheless, no ORs differed from unity to a statistically significant degree, and there were no consistent dose–response relationships with pack-years or smoking duration.

**Table 4:** Odds ratios between smoking and brain tumour by sex, ever regular smoking, smoking duration and pack-years

**Sex/smoking stratum**	**Glioma**		**Meningioma**	
	**Ca/Co**	**OR (95% CI)**	**Ca/Co**	**OR (95% CI)**
***Males and females combined***				
Never regular smokers	78/311	1	42/311	1
Ever regular smokers	88/337	0.96 (0.67-1.38)	51/337	1.09 (0.69-1.72)
>0 to <20 years	51/177	1.05 (0.70-15.96)	25/177	1.10 (0.64-1.89)
≥ 20 years	37/160	0.84 (0.53-1.34)	26/160	1.08 (0.62-1.88)
>0 to <15 pack-years	43/173	0.97 (0.63-1.49)	22/173	0.93 (0.53-1.62)
≥15 pack-years	45/164	0.95 (0.61-1.48)	29/164	1.28 (0.75-2.20)
***Males only***				
Never regular smokers	41/140	1	9/140	1
Ever regular smokers	64/177	1.28 (0.82-2.02)	17/177	1.34 (0.57-3.17)
>0 to <20 years	37/88	1.36 (0.80-2.30)	16/89	1.47 (0.56-3.90)
≥ 20 years	27/89	1.02 (0.57-1.82)	18/71	1.21 (0.44-3.36)
>0 to <15 pack-years	30/75	1.32 (0.75-2.30)	17/98	1.00 (0.32-3.11)
≥15 pack-years	34/102	1.10 (0.63-1.89)	17/62	1.61 (0.63-4.13)
***Females only***				
Never regular smokers	37/171	1	33/171	1
Ever regular smokers	24/160	0.73 (0.41-1.28)	34/160	0.98 (0.57-1.68)
>0 to <20 years	14/89	0.78 (0.40-1.53)	9/88	0.99 (0.51-1.92)
≥ 20 years	10/71	0.66 (0.31-1.42)	8/89	0.98 (0.50-1.89)
>0 to <15 pack-years	13/98	0.68 (0.34-1.36)	5/75	0.93 (0.48-1.78)
≥15 pack-years	11/62	0.78 (0.37-1.65)	12/102	1.05 (0.53-2.08)

Three models were estimated: one model with ever regular smoking as a binary variable and one each with three categorical levels of duration and pack-years of smoking. The reference groups in all comparisons are never regular smokers. Analyses were adjusted for age (decades); sex (in the analysis of males and females combined); education level (low, high); and region (Montreal, Ottawa, Vancouver). Covariates were removed from the model if they were non-significant and did not change any of the smoking ORs by more than 10%.

Because the use of proxy response for subjects who were too ill or deceased could have introduced error or bias in the data, we carried out analyses analogous to those in Table 4, but restricted to self-respondents only. There were no important differences in ORs between the two sets of results (results not shown).

We next examined second-order models with potential interaction terms or effect modifiers of the relationship between ever regular smoking and glioma and meningioma. For glioma, there was no statistically significant evidence of effect modification by sex, age, or other potential effect modifier, except that among males there was a significant interaction between ever regular smoking and education level (p = 0.032). Among females, the interaction was suggestive but did not reach statistical significance (p = 0.066). These results are shown in Table 5. The OR between ever regular smoking and glioma among males and females combined was 1.33 (95% CI: 0.62 – 2.85) in the low education stratum and 0.48 (95% CI: 0.25 – 0.91) in the high education stratum. The apparent interaction between smoking and education level was especially striking among males. In order to evaluate the possibility that smokers with lower education may have been heavier smokers, we added pack years to the model. It was not statistically significant and did not meaningfully change the ORs of the ever regular smoking variable or the ever regular smoking-education interaction. Finally, while interaction terms for ever smoking and sex were not statistically significant at the alpha = 0.05 level, Table 5 shows noteworthy differences in ever regular smoking OR by sex.

**Table 5:** Odds ratios between ever versus never regular smoking for glioma by education level

**Sex/education stratum**	**Glioma**	
	**Ca/Co**	**OR (95% CI)**
***Males and females combined***		
Ever regular smoking-education interaction p = 0.010		
Ever versus never regular smoking, lower education	64/186	1.33 (0.62-2.85)
Ever versus never regular smoking, higher education	102/462	0.48 (0.25-0.91)
***Males only***		
Ever regular smoking-education interaction p = 0.032		
Ever versus never regular smoking, lower education	39/87	2.79 (1.08-7.18)
Ever versus never regular smoking, higher education	66/230	0.85 (0.49-1.48)
***Females only***		
Ever regular smoking-education interaction p = 0.066		
Ever versus never regular smoking, lower education	25/99	1.31 (0.53-3.22)
Ever versus never regular smoking, higher education	36/232	0.42 (0.19-0.95)

Models were of ever regular smoking as a binary variable, with the addition of an interaction term of ever regular smoking and education level. The reference groups in all comparisons are never regular smokers in the appropriate sex and education stratum. Analyses were adjusted for age (decades); sex (in the analysis of males and females combined); and region (Montreal, Ottawa, Vancouver). Covariates were removed from the model if they were non-significant and did not change any of the smoking ORs by more than 10%.

For meningioma, there was no statistically significant evidence of effect modification of ever regular smoking by age, sex, education or study centre (results not shown), and none that resulted in a statistically significant elevation in OR for ever smoking.

## Discussion

Cigarette smoke, a complex mixture of chemical substances, is the single most important cause of human cancer. It has already been shown to induce tumours in numerous organs and tissues. While the carcinogenicity of smoking is not in doubt and the recommendation to reduce or eliminate smoking in society would hardly be affected by the identification of new tumour sites affected by smoking, the investigation of a possible link between smoking and brain tumours is important for our understanding of brain tumour aetiology.

While it remains uncertain whether carcinogens from tobacco smoke, such as PAHs and *N*-nitroso compounds, might reach brain tissue, animal experimentation suggests that *N*-nitroso compounds are potent animal nervous system carcinogens. However, previous epidemiologic research has not provided a clear indication of whether smoking can increase the risk of brain tumours. While most studies have failed to demonstrate any excess risks [5,13-20,25-27,30-32], some studies have found excess risks in their entire population or in some subpopulation [28,29,34,35].

The present study did not demonstrate an overall association between ever regular smoking and either glioma or meningioma, nor was there convincing evidence of an exposure-response relationship between smoking and either of these lesions. The only exception to a consistent pattern of null results for smoking and brain tumours was the unexpected finding of a higher OR between ever regular smoking and glioma among subjects with lower education compared to that among subjects with higher education. This resulted in a statistically significant elevation in the OR for ever regular smokers among males with lower education. However, this was accompanied by a statistically significant deficit of risk among females with higher education. In other words, the excess risk due to smoking among the lower educated was concentrated among males, while the deficit of risk due to smoking among the higher educated was concentrated among females. The participation proportions were lower among controls than among cases. While low participation proportions do not necessarily imply bias, it is possible that participating controls may have been unrepresentative of all eligible control subjects, at least for education level. Our comparison of subjects eligible to be controls found that participants tended to have higher educational attainment than non-participants. Our comparison of smoking measures among controls with lower and higher educational attainment found that those with lower attainment were more likely to have ever smoked and the smokers among them smoked more heavily. It is therefore possible that subjects with lower education and greater smoking were under-represented in our control group. We examined the possible effect of smoking intensity on the risk associated with ever regular smoking by adding pack years to the ever regular smoking model for both sexes together and for males and females separately. In none of these models was pack years statistically significant, nor did it meaningfully alter the ORs for ever regular smoking or for the interaction between ever smoking and education. Regarding the sex distribution of our samples, while the proportion of males among controls was lower than that among glioma cases and higher than that among meningioma cases, similar interaction effects were seen in males and females together and separately. Whether the pattern of interactions we report was due to a real effect modification of smoking by education and sex or to some combination of chance and bias is difficult to answer from our dataset. There are some features that argue against a causal interpretation, including the following: there was no prior hypothesis for such interactions; they arose in a multiple testing set of subgroup analyses; they would imply an implausible protective effect of smoking in one part of the population [39]; there was no clear dose–response relationship demonstrated; and low participation rates raise the possibility of selection biases which might have unusual subgroup patterns. Before engaging in speculation on possible biological pathways to explain possible effect modifications by sex or education, both of which are proxies for complex sets of environmental, social and biological variables, it would be useful if other investigators would examine whether such interactions can be detected in other datasets.

Our study had several strengths. It was based on well-substantiated cases from three Canadian regions, a large population control series, and detailed lifetime smoking histories obtained via face-to-face interviews by trained interviewers. It is generally considered that smoking is reported with reasonable accuracy, and, since smoking is not generally considered to be a cause of brain tumours, it is unlikely that cases and controls would have differentially reported their smoking histories. It is unlikely that the use of proxy respondents for some subjects induced bias, since parallel analyses restricted to self-report subjects yielded ORs similar to those shown.

The main limitations included the limited numbers of cases and the relatively low response rates in some subgroups. Difficulty in recruitment is becoming a problem of increasing importance in epidemiologic studies, including the INTERPHONE study [38,40,41], and our participation proportions are consistent with this trend. Theoretically, depending on the true but unknown smoking patterns among nonparticipants, it would be possible that nonrepresentative control series could have masked a true overall effect or created spurious effects in some subgroups. Unfortunately, without data on smoking patterns among nonparticipants, we cannot infer whether this did or did not bias our estimated ORs. However, one indirect index of overall representativeness comes from the comparison of smoking rates in our control group with published national data on smoking rates in Canada [42]. The fraction of current smokers reported by our participant control group was 22.2% overall, 24.6% among males and 19.9% among females. This is similar to 2003 national Canadian averages for the 35–64 year age range of 25.2% overall, 27.2% among males and 23.2% among females [43]. Because of different study areas between the national survey and our investigation, different sampling schemes and different instruments, we cannot expect identical estimates, but the national data do provide a useful benchmark for judging the representativeness of our participants.

A final caveat pertains to the fact that our study subjects were 30 to 59 years of age. It is conceivable that there might be a long latency period for tobacco-induced brain tumours, and that consequently any risk due to smoking would only be detectable in older brain tumour cases. Our data are therefore only informative regarding possible risks that manifest before the age of 60.

## Conclusion

Our study did not demonstrate a significant overall excess risk of glioma or meningioma with smoking. Given the limited numbers of cases, however, we cannot affirm with confidence that our results demonstrate an absence of a true effect. Subgroup analyses indicated excess risks of glioma due to smoking among males with lower educational attainment, accompanied by a decreased risk due to smoking among females with high educational attainment. This statistically significant interaction between ever smoking, sex and education may merit further exploration.

### Financial support

The Montreal data collection was funded by a grant from the Canadian Institutes of Health Research (project MOP-42525) and the analysis was funded by the Guzzo-SRC Chair in Environment and Cancer. Dr. Siemiatycki had salary support from the Canada Research Chair programme. Dr. Parent had salary support from the Fonds de recherche en santé du Québec. Dr. Abrahamowicz is supported by the James McGill Chair at McGill University. The Ottawa and Vancouver centres were supported by a university-industry partnership grant from the Canadian Institutes of Health Research (CIHR), the latter including partial support from the Canadian Wireless Telecommunications Association. The CIHR university-industry partnerships program also includes provisions that ensure complete scientific independence of the investigators. Dr. Krewski is the Natural Sciences and Engineering Research Council of Canada Chair in Risk Science at the University of Ottawa. Coordination of the INTERPHONE study and development of the study instruments were supported by funding from the European Fifth Framework Program, “Quality of Life and Management of Living Resources” [contract QLK4-CT-1999901563] and the International Union against Cancer (UICC). The UICC received funds for this purpose from the Mobile Manufacturers’ Forum and GSM Association. Provision of funds to the INTERPHONE study investigators via the UICC was governed by agreements that guaranteed INTERPHONE's complete scientific independence. The terms of these agreements are publicly available at http://www.interphone.iarc.fr.

## Competing interests

The authors declare that they have no competing interests.

## Authors’ contributions

SV performed the data analysis and wrote the manuscript. EC was principle investigator of the international INTERPHONE study and contributed to the analysis of the present study. LR, JS, DK, MM, KL, and M-EP contributed to the local implementation of the international INTERPHONE study design. LR was coordinator for the international INTERPHONE study, was responsible for quality control of data collection, contributed to questionnaire development, and provided assistance with data management and analysis. JS, DK and MM were principal investigators of the international INTERPHONE study at the Montreal, Ottawa and Vancouver sites, respectively. KL provided assistance with the parameterization of smoking variables and with modeling strategy. M-EP was participating author of the INTERPHONE study at the Montreal site and provided assistance with data analysis. MA provided statistical advice and assistance with data analysis. All authors reviewed and edited the manuscript.

## References

[B1] Fisher JL Schwartzbaum JA Wrensch M Wiemels JL Epidemiology of brain tumors Neurol Clin 2007 25 4 867 890 vii 10.1016/j.ncl.2007.07.002 17964019

[B2] Ohgaki H Kleihues P Epidemiology and etiology of gliomas Acta Neuropathol 2005 109 1 93 108 10.1007/s00401-005-0991-y 15685439

[B3] Wrensch M Fisher JL Schwartzbaum JA Bondy M Berger M Aldape KD The molecular epidemiology of gliomas in adults Neurosurg Focus 2005 19 5 E5 16398469 10.3171/foc.2005.19.5.6

[B4] Marosi C Hassler M Roessler K Reni M Sant M Mazza E Vecht C Meningioma Crit Rev Oncol Hematol 2008 67 2 153 171 10.1016/j.critrevonc.2008.01.010 18342535

[B5] Zheng T Cantor KP Zhang Y Chiu BC Lynch CF Risk of brain glioma not associated with cigarette smoking or use of other tobacco products in Iowa Cancer Epidemiol Biomarkers Prev 2001 10 4 413 414 11319186

[B6] Preston-Martin S Yu MC Benton B Henderson BE N-Nitroso compounds and childhood brain tumors: a case–control study Cancer Res 1982 42 12 5240 5245 7139628

[B7] Maekawa A Mitsumori K Spontaneous occurrence and chemical induction of neurogenic tumors in rats–influence of host factors and specificity of chemical structure Crit Rev Toxicol 1990 20 4 287 310 10.3109/10408449009089866 2178628

[B8] Bogovski P Bogovski S Animal Species in which N-nitroso compounds induce cancer Int J Cancer 1981 27 4 471 474 10.1002/ijc.2910270408 7275353

[B9] Hawkins BT Egleton RD Davis TP Modulation of cerebral microvascular permeability by endothelial nicotinic acetylcholine receptors Am J Physiol Heart Circ Physiol 2005 289 1 H212 H219 10.1152/ajpheart.01210.2004 15708958

[B10] Connelly JM Malkin MG Environmental risk factors for brain tumors Curr Neurol Neurosci Rep 2007 7 3 208 214 10.1007/s11910-007-0032-4 17488586

[B11] Irigaray P Newby JA Clapp R Hardell L Howard V Montagnier L Epstein S Belpomme D Lifestyle-related factors and environmental agents causing cancer: an overview Biomed Pharmacother 2007 61 10 640 658 10.1016/j.biopha.2007.10.006 18055160

[B12] Smoking and cancer [http://www.cancer.ca/en/prevention-and-screening/live-well/smoking-and-tobacco/smoking-and-cancer/?region=qc]

[B13] Preston-Martin S Mack W Henderson BE Risk factors for gliomas and meningiomas in males in Los Angeles county Cancer Res 1989 49 21 6137 6143 2790826

[B14] Brownson RC Reif JS Chang JC Davis JR An analysis of occupational risks for brain cancer Am J Public Health 1990 80 2 169 172 10.2105/AJPH.80.2.169 2297060 PMC1404624

[B15] Hochberg F Toniolo P Cole P Salcman M Nonoccupational risk indicators of glioblastoma in adults J Neurooncol 1990 8 1 55 60 2319291 10.1007/BF00182087

[B16] Schlehofer B Kunze S Sachsenheimer W Blettner M Niehoff D Wahrendorf J Occupational risk factors for brain tumors: results from a population-based case–control study in Germany Cancer Causes Control 1990 1 3 209 215 10.1007/BF00117472 2102293

[B17] Preston-Martin S Mack W Gliomas and meningiomas in men in Los Angeles county: investigation of exposures to N-nitroso compounds IARC Sci Publ 1991 105 197 203 1855850

[B18] Ryan P Lee MW North B McMichael AJ Risk factors for tumors of the brain and meninges: results from the Adelaide adult brain tumor study Int J Cancer 1992 51 1 20 27 10.1002/ijc.2910510105 1563840

[B19] Blowers L Preston-Martin S Mack WJ Dietary and other lifestyle factors of women with brain gliomas in Los Angeles county (California, USA) Cancer Causes Control 1997 8 1 5 12 10.1023/A:1018437031987 9051317

[B20] Schlehofer B Hettinger I Ryan P Blettner M Preston-Martin S Little J Arslan A Ahlbom A Giles GG Howe GR Ménégoz F Rodvall Y Choi WN Wahrendorf J Occupational risk factors for low grade and high grade glioma: results from an international case control study of adult brain tumours Int J Cancer 2005 113 1 116 125 10.1002/ijc.20504 15386358

[B21] Musicco M Filippini G Bordo BM Melotto A Morello G Berrino F Gliomas and occupational exposure to carcinogens: case–control study Am J Epidemiol 1982 116 5 782 790 7148804 10.1093/oxfordjournals.aje.a113468

[B22] Ahlbom A Navier IL Norell S Olin R Spannare B Nonoccupational risk indicators for astrocytomas in adults Am J Epidemiol 1986 124 2 334 337 3728449 10.1093/oxfordjournals.aje.a114393

[B23] Hurley SF McNeil JJ Donnan GA Forbes A Salzberg M Giles GG Tobacco smoking and alcohol consumption as risk factors for glioma: a case–control study in Melbourne J Epidemiol Community Health 1996 50 4 442 446 10.1136/jech.50.4.442 8882229 PMC1060316

[B24] Lee M Wrensch M Miike R Dietary and tobacco risk factors for adult onset glioma in the San Francisco Bay Area (California, USA) Cancer Causes Control 1997 8 1 13 24 10.1023/A:1018470802969 9051318

[B25] Mills PK Preston-Martin S Annegers JF Beeson WL Phillips RL Fraser GE Risk factors for tumors of the brain and cranial meninges in Seventh-Day Adventists Neuroepidemiology 1989 8 5 266 275 10.1159/000110193 2812186

[B26] Holick CN Giovannucci EL Rosner B Stampfer MJ Michaud DS Prospective study of cigarette smoking and adult glioma: dosage, duration, and latency Neuro Oncol 2007 9 3 326 334 10.1215/15228517-2007-005 17504930 PMC1907415

[B27] Benson VS Pirie K Green J Casabonne D Beral V Lifestyle factors and primary glioma and meningioma tumours in the Million Women Study cohort Br J Cancer 2008 99 1 185 190 10.1038/sj.bjc.6604445 18560401 PMC2453038

[B28] Efird JT Friedman GD Sidney S Klatsky A Habel LA Udaltsova NV Van den Eeden S Nelson LM The risk for malignant primary adult-onset glioma in a large, multiethnic, managed-care cohort: cigarette smoking and other lifestyle behaviors J Neurooncol 2004 68 1 57 69 15174522 10.1023/b:neon.0000024746.87666.ed

[B29] Silvera SA Miller AB Rohan TE Cigarette smoking and risk of glioma: a prospective cohort study Int J Cancer 2006 118 7 1848 1851 10.1002/ijc.21569 16217772

[B30] Mandelzweig L Novikov I Sadetzki S Smoking and risk of glioma: a meta-analysis Cancer Causes Control 2009 20 10 1927 1938 10.1007/s10552-009-9386-z 19568697

[B31] Schneider B Pulhorn H Rohrig B Rainov NG Predisposing conditions and risk factors for development of symptomatic meningioma in adults Cancer Detect Prev 2005 29 5 440 447 10.1016/j.cdp.2005.07.002 16188400

[B32] Lee E Grutsch J Persky V Glick R Mendes J Davis F Association of meningioma with reproductive factors Int J Cancer 2006 119 5 1152 1157 10.1002/ijc.21950 16570277

[B33] Preston-Martin S Yu MC Henderson BE Roberts C Risk factors for meningiomas in men in Los Angeles County J Natl Cancer Inst 1983 70 5 863 866 6573530

[B34] Hu J Little J Xu T Zhao X Guo L Jia X Huang G Bi D Liu R Risk factors for meningioma in adults: a case–control study in northeast China Int J Cancer 1999 83 3 299 304 10.1002/(SICI)1097-0215(19991029)83:3<299::AID-IJC2>3.0.CO;2-Z 10495419

[B35] Phillips LE Longstreth WT Jr Koepsell T Custer BS Kukull WA van Belle G Active and passive cigarette smoking and risk of intracranial meningioma Neuroepidemiology 2005 24 3 117 122 10.1159/000082998 15637448

[B36] Batty GD Kivimaki M Gray L Davey Smith G Marmot MG Shipley MJ Cigarette smoking and site-specific cancer mortality: testing uncertain associations using extended follow-up of the original Whitehall study Ann Oncol 2008 19 5 996 1002 10.1093/annonc/mdm578 18212091

[B37] International Agency for Research in Cancer (IARC) Tobacco Smoking IARC Monographs, A Review of Human Carcinogens: Personal Habits and Indoor Combustions 2012 Lyon: IARC Press 100E: 43-211

[B38] Cardis E Richardson L Deltour I Armstrong B Feychting M Johansen C Kilkenny M McKinney P Modan B Sadetzki S Schüz J Swerdlow A Vrijheid M Auvinen A Berg G Blettner M Bowman J Brown J Chetrit A Christensen HC Cook A Hepworth S Giles G Hours M Iavarone I Jarus-Hakak A Klaeboe L Krewski D Lagorio S Lönn S The INTERPHONE study: design, epidemiological methods, and description of the study population Eur J Epidemiol 2007 22 9 647 664 10.1007/s10654-007-9152-z 17636416

[B39] Weiss NS Subgroup-specific associations in the face of overall null results: should we rush in or fear to tread? Cancer Epidemiol Biomarkers Prev 2008 17 6 1297 1299 10.1158/1055-9965.EPI-08-0144 18559544

[B40] Chen J Wacholder S Morton LM Bhatti P Hartge P Quantifying selection bias in epidemiologic studies Am J Epidemiol 2005 161 Suppl S145

[B41] Hartge P Participation in population studies Epidemiology 2006 17 3 252 254 10.1097/01.ede.0000209441.24307.92 16617271

[B42] Millar WJ Reaching smokers with lower educational attainment Statistics Canada Health Reports 1996 Ottawa: Statistics Canada Health Analysis Division 8(2):11-19(Eng); 13-22(Fre) 9110961

[B43] Shields M Smoking-prevalence, bans and exposure to second-hand smoke Health Rep 2007 18 3 67 85 17892252

